# First Clinical Application and Validation of the Romanian BREAST-Q in Immediate and Delayed Breast Reconstruction: A Prospective Study

**DOI:** 10.3390/cancers18010168

**Published:** 2026-01-03

**Authors:** Andrada-Elena Ţigăran, Adelaida Avino, Abdalah Abu-Baker, Teodora Timofan, Daniela-Elena Ion, Daniela-Elena Gheoca-Mutu, Radu-Cristian Jecan, Erick George Neștianu, Laura Raducu

**Affiliations:** 1 Doctoral School, “Carol Davila” University of Medicine and Pharmacy, 010221 Bucharest, Romania; andrada-elena.tigaran@drd.umfcd.ro (A.-E.Ţ.); abdalah.abu-baker@drd.umfcd.ro (A.A.-B.); 2 Discipline of Plastic Surgery, “Carol Davila” University of Medicine and Pharmacy, 010221 Bucharest, Romania; teodora.peligrad@rez.umfcd.ro (T.T.); daniela-elena.ion@rez.umfcd.ro (D.-E.I.); cristian.jecan@umfcd.ro (R.-C.J.); laura.raducu@umfcd.ro (L.R.); 3 Department of Plastic Surgery, Agrippa Ionescu Emergency Clinical Hospital, 011356 Bucharest, Romania; daniela-elena.mutu@umfcd.ro; 4 Discipline of Anatomy, “Carol Davila” University of Medicine and Pharmacy, 010221 Bucharest, Romania; 5 Department of Radiology, Agrippa Ionescu Emergency Clinical Hospital, 011356 Bucharest, Romania; nestianuerick@gmail.com

**Keywords:** breast cancer, breast reconstruction, quality of life, psychosocial impact, BREAST-Q, immediate breast reconstruction, autologous and implant-based reconstruction

## Abstract

**Objectives:** Breast cancer treatment has increasingly shifted toward integrating patient-reported outcomes into surgical decision-making. However, Romania has lacked a validated instrument to evaluate quality of life (QoL) after breast reconstruction. This study aimed to translate, culturally adapt, and clinically validate the Romanian version of the BREAST-Q Reconstruction Module, and to assess postoperative changes in QoL following immediate and delayed breast reconstruction. **Methods:** A prospective observational study enrolled 116 patients undergoing immediate or delayed implant-based or autologous reconstruction between June 2023 and June 2024. The BREAST-Q was translated using standardized forward–backward methodology, followed by expert review, pilot testing, and psychometric validation. Patients completed preoperative and 1-year postoperative BREAST-Q questionnaires. Statistical analyses included Cronbach’s α, intraclass correlation coefficients, paired and independent *t*-tests, Pearson correlations, and ANCOVA to assess the impact of radiotherapy and other clinical factors on postoperative outcomes. **Results:** The Romanian BREAST-Q showed excellent internal consistency (α = 0.947) and strong test–retest reliability (ICC = 0.81–0.92). Both immediate and delayed reconstruction significantly improved psychosocial well-being and breast symptoms (p < 0.001). Immediate reconstruction produced modest gains in physical well-being, whereas delayed reconstruction yielded a substantial increase in breast satisfaction (effect size d = 1.33). Breast sensation significantly decreased in both cohorts. Within delayed reconstructions, deep inferior epigastric perforator (DIEP) flaps were associated with higher physical well-being, whereas latissimus dorsi (LD) flaps demonstrated slightly better sensory outcomes. Radiotherapy had a strong negative effect on postoperative breast satisfaction (p < 0.001) after adjusting for covariates. **Conclusions:** The Romanian BREAST-Q is a valid, reliable instrument for evaluating QoL after breast reconstruction. Reconstruction improved multiple QoL domains, although sensory decline persisted across techniques. Symmetrization and DIEP reconstruction enhanced postoperative comfort, while radiotherapy remained a major predictor of lower satisfaction. These findings support the need for integrative reconstructive strategies that address aesthetic, functional, and sensory outcomes.

## Simple Summary

Breast reconstruction is an integral component of modern breast cancer surgery, aiming to restore body image and quality of life alongside oncologic treatment. Patient-reported outcome measures are essential for understanding the impact of reconstructive procedures from the patient’s perspective; however, until now, no validated Romanian instrument was available for this purpose. This study translated, culturally adapted, and clinically validated the Romanian version of the BREAST-Q Reconstruction Module. A total of 116 women completed the questionnaire before and one year after immediate or delayed implant-based or autologous breast reconstruction. The Romanian BREAST-Q demonstrated excellent reliability and sensitivity to postoperative change. Both immediate and delayed reconstruction were associated with significant improvements in psychosocial well-being and breast-related symptoms. Radiotherapy negatively affected postoperative satisfaction, while symmetrization procedures were associated with greater physical comfort. These findings support the use of the Romanian BREAST-Q for assessing patient-reported outcomes and highlight the role of breast reconstruction in improving quality of life within contemporary breast cancer care.

## 1. Introduction

Quality of life (QoL) is a multidimensional concept that determines an individual’s well-being at a given moment [1]. In the early 1970s, the QoL concept began to be discussed as a measure of well-being in patients with various illnesses or disabilities. Since then, interest in assessing QoL has grown considerably, and the World Health Organization has emphasized its importance at all stages of life [2,3]. QoL is closely related to each person’s goals, expectations, standards, and living environment [4].

The significant increase in breast cancer incidence has led to major advances in its diagnosis and treatment, greatly improving patient survival rates. However, beyond survival, current studies emphasize the QoL of patients diagnosed with breast neoplasms [4].

Beyond concerns related to diagnosis and recurrence, breast cancer is associated with significant physical, psychological, and social consequences that can adversely affect quality of life, including changes in family dynamics, mental health, and sexual well-being [5,6,7].

The choice of surgical treatment significantly influences quality of life in patients with breast cancer. In Europe, breast-conserving surgery represents the preferred approach for early-stage disease and is performed more frequently than total mastectomy, reflecting advances in screening, systemic therapies, and oncoplastic techniques. According to Eurostat data from 2021, substantial variation exists across European countries regarding surgical practice patterns. In Romania, the rate of partial excision of the mammary gland was 21.7 per 100,000 inhabitants, while the rate of total mastectomy was 20.2 per 100,000 inhabitants [8]. These differences likely reflect variations in national screening programs, stage at diagnosis, healthcare infrastructure, and access to reconstructive services.

Mastectomy is considered one of the most psychologically devastating therapeutic surgical management methods. This procedure affects self-esteem, femininity, and body image, causing more trauma than cancer itself [9].

Evaluating QoL in patients diagnosed with breast cancer is essential and improving it should be one of the main objectives within breast cancer treatment protocols [10].

Current studies indicate that breast reconstruction can improve QoL through its positive impact on body image, self-perception, and emotional health, reducing feelings of disfigurement. However, the psychological implications of reconstructive surgery can vary greatly from patient to patient. The subjective nature of QoL remains a complex and multifactorial issue. The timing and type of reconstruction, the patient’s individual characteristics, and psychosocial support are determining factors of the overall impact on QoL [11].

Several generic and disease-specific instruments are available to assess health-related quality of life; however, the BREAST-Q is the most widely used tool for evaluating patient-reported outcomes after mastectomy and breast reconstruction. Developed specifically for breast surgery, the BREAST-Q includes preoperative and postoperative modules that assess quality of life and patient satisfaction and is therefore recommended for evaluating the impact of breast surgical interventions [12,13,14].

In Romania, the evaluation of QoL in breast cancer patients who have undergone oncologic resection and breast reconstruction has not been specifically carried out, due to the lack of a measurement tool such as BREAST-Q.

This study aims to evaluate the QoL of patients undergoing breast reconstruction using the BREAST-Q questionnaire, now translated and validated in Romanian, being the first national study to analyze the positive impact of reconstructive surgery.

## 2. Materials and Methods

To ensure accurate cultural adaptation and psychometric validity of the BREAST-Q for Romanian-speaking patients, we followed internationally accepted guidelines for cross-cultural adaptation of patient-reported outcome measures [15]. First, two independent bilingual translators (fluent in English and native Romanian speakers) performed **forward translations** of the original BREAST-Q modules. These versions were synthesized into a single preliminary Romanian version by an expert committee. A **back-translation** into English was then carried out by two separate translators blinded to the original instrument to verify semantic and conceptual equivalence.

An expert panel of breast reconstructive surgeons, oncologists, psychologists, and linguists reviewed all the versions to resolve discrepancies and ensure clinical relevance, cultural appropriateness, and linguistic clarity. A **pilot test** was subsequently conducted with a small cohort of post-mastectomy and breast reconstruction patients (n = 10) to assess comprehension, acceptability, and item clarity. Based on patient feedback and panel review, minor adjustments were made. Finally, **psychometric validation** was performed in a larger sample (n = 25) to assess internal consistency (Cronbach’s α), test–retest reliability (intraclass correlation coefficient; n = 12), construct validity, and responsiveness, and floor and ceiling effects were evaluated using the 15% threshold. The sample size was considered adequate for psychometric validation analyses, in line with methodological recommendations for questionnaire validation studies, which suggest a minimum of 5–10 participants per questionnaire item or a total sample size exceeding 100 subjects for reliability and internal consistency assessment

Internal consistency reliability of the Romanian BREAST-Q subscales was evaluated using **Cronbach’s alpha (α)**. An α value ≥ 0.70 was considered acceptable, ≥0.80 good, and ≥0.90 excellent. Calculations were performed for each BREAST-Q domain (satisfaction with breasts, psychosocial well-being, sexual well-being, physical well-being, satisfaction with nipple areola complex, satisfaction with abdomen, animation deformity, breast sensation, breast symptoms, sensation: QoL impact, back appearance, physical well-being: back and shoulder and adverse effects of radiation) using baseline responses from the study cohort.

Test–retest reliability was assessed in a subgroup of 12 patients who completed the BREAST-Q twice, two weeks apart. Intraclass correlation coefficients (ICC, two-way mixed, absolute agreement) were calculated for each domain.

We conducted a prospective observational study at the Department of Plastic and Reconstructive Surgery in” Agrippa Ionescu” Emergency Clinical Hospital, enrolling 116 patients who underwent breast reconstruction between **June 2023 and June 2024**. The study was approved by the institutional ethics committee (approval no. 104663/10.04.2020), and all participants provided written informed consent prior to inclusion.

**Figure 1:**
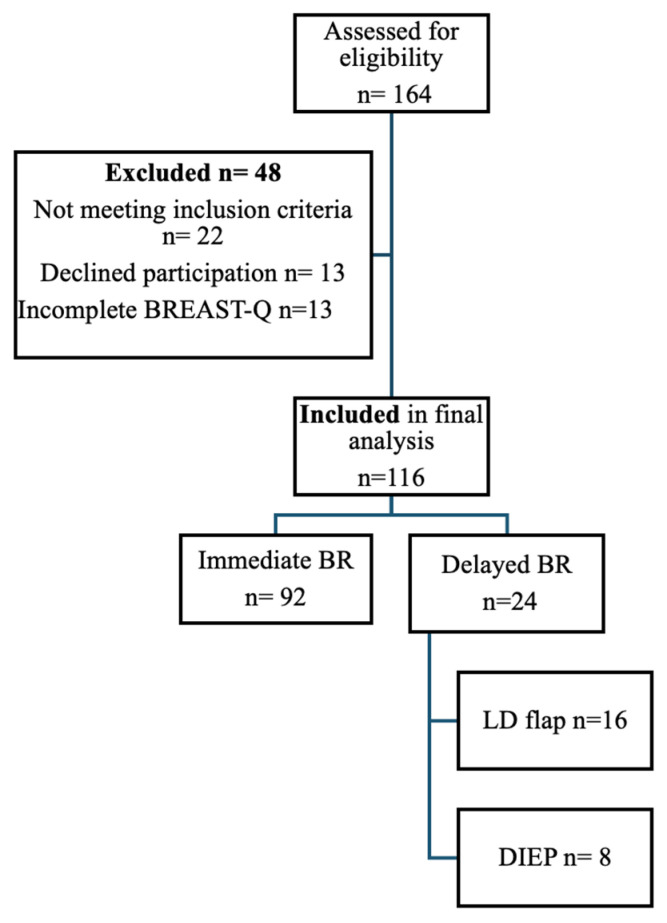
CONSORT-style flow diagram illustrating patient selection in the study.

Eligible participants were adult female patients (≥18 years) who underwent therapeutic or risk-reducing mastectomy followed by immediate or delayed breast reconstruction at our institution and were able to complete the validated Romanian version of the BREAST-Q Reconstruction Module. Inclusion required written informed consent and availability for postoperative assessment at approximately 12 months. Patients undergoing aesthetic breast procedures, revision-only surgeries, prior breast reconstruction outside the study period, incomplete BREAST-Q assessments, withdrawal of consent, or advanced recurrent or metastatic disease precluding meaningful follow-up were excluded. Patient selection is presented in a CONSORT-style flow diagram [Figure 1].

Patient-reported outcomes were assessed using the BREAST-Q Reconstruction Module (preoperative and 1-year postoperative time points). The preoperative questionnaire was administered within 2 weeks prior to surgery (before mastectomy and direct reconstruction or before breast reconstruction for the delayed group). The postoperative questionnaire was completed at approximately 12 months (±2 months) after the reconstruction. Patients within the delayed reconstruction group were referred to us from other clinics.

Domains analyzed included: satisfaction with breasts, psychosocial well-being, sexual well-being, physical well-being, satisfaction with nipple areola complex (NAC), satisfaction with abdomen, animation deformity, breast sensation, breast symptoms, sensation: QoL impact, back appearance, physical well-being: back and shoulder and adverse effects of radiation.

Clinical data (age, comorbidities, type of reconstruction, timing of reconstruction, adjuvant therapies) were collected from electronic medical records.

BREAST-Q raw responses were converted into 0–100 scale scores according to the Q-Score manual guidelines, where higher scores indicate greater satisfaction or better QoL.

Data were analyzed using IBM SPSS Statistics (version 31; IBM Corp., Armonk, NY, USA). Descriptive statistics were calculated for all variables and presented as means ± standard deviation (SD) or frequencies and percentages, as appropriate. Statistical analyses were predefined according to the primary objective of the study, which was the psychometric validation of the Romanian version of the BREAST-Q Reconstruction Module. Planned analyses included assessment of internal consistency (Cronbach’s α), test–retest reliability (intraclass correlation coefficient), and convergent validity. Comparisons between clinical and reconstructive subgroups, as well as correlation analyses between BREAST-Q domains and clinical variables, were performed as **exploratory analyses**. Internal consistency of the Romanian BREAST-Q was assessed using Cronbach’s α, and test–retest reliability was evaluated with ICC, two-way mixed-effects model, absolute agreement. Paired samples *t*-tests were applied to compare preoperative and postoperative BREAST-Q scores within groups, while independent samples *t*-tests were used for between-group comparisons (e.g., immediate vs. delayed reconstruction; deep inferior epigastric perforator flap (DIEP) vs. latissimus dorsi flaps (LD)). Effect sizes were reported using Cohen’s d, and equality of variances was verified using Levene’s test. Associations between continuous variables were explored using Pearson’s correlation coefficients, effect sizes were interpreted using established thresholds, with correlation coefficients categorized as very strong (r = 0.80–1.00), strong (0.60–0.79), moderate (0.40–0.59), weak (0.20–0.39), and very weak (0.00–0.19), and visualized with a correlation heatmap. To examine the influence of clinical variables (e.g., symmetrization, radiotherapy) on postoperative outcomes while controlling for covariates, analyses of covariance (ANCOVA) were performed. Statistical significance was set at p < 0.05 for all tests.

## 3. Results

### 3.1. Psychometric Validation of the Romanian BREAST-Q

The Romanian BREAST-Q version demonstrated **excellent internal consistency** across its 16 items, with an overall **Cronbach’s alpha of 0.947**. The instrument demonstrated good to excellent internal consistency across all quality-of-life domains, including Psychosocial Well-Being (α = 0.96), Physical Well-Being: Chest (α = 0.93), Sexual Well-Being (α = 0.89), Satisfaction with Breasts (α = 0.91), Breast Symptoms (α = 0.95), and Satisfaction with Outcome (α = 0.89). Among patients undergoing autologous reconstruction, the Physical Well-Being: Abdomen domain also showed good internal consistency (α = 0.92). Care-related domains—including Satisfaction with Information (α = 0.98), Satisfaction with Surgeon (α = 0.95), Satisfaction with Medical Team (α = 0.90), and Satisfaction with Office Staff (α = 0.88)—likewise demonstrated high reliability. No relevant floor or ceiling effects were observed across BREAST-Q domains, as fewer than 15% of respondents achieved minimum or maximum scores in any domain. Corrected item–total correlations ranged from 0.362 to 0.971, indicating that most items were strongly associated with the total score—Table 1 Examination of the “Cronbach’s Alpha if Item Deleted” column showed minimal variation (range: 0.938–0.952), suggesting that removal of any single item would not meaningfully improve the scale’s reliability. These findings confirm the robust internal consistency and stability of the translated instrument for assessing QoL and satisfaction among Romanian breast reconstruction patients.

**Test–retest reliability** was evaluated in a subgroup of 12 clinically stable patients who completed the BREAST-Q twice, two weeks apart. ICC, two-way mixed-effects model, absolute agreement ranged from 0.81 to 0.92 across all domains, confirming good to excellent stability of the Romanian BREAST-Q over time.

### 3.2. Descriptive Characteristics of the Study Cohort

The study cohort included **116 patients** who underwent breast reconstruction in our clinic between 01.06.2023 and 01.06.2024. The mean age of the patients was 47.9 years (SD = 10.16), with individuals ranging from 33 to 70 years old. The length of hospital stay averaged 7.76 days (SD = 3.04), with a minimum stay of 4 days and a maximum of 15 days. The longer length of hospital stay reflects institutional discharge practices and patient-related logistical factors, including geographic distance and limited access to early outpatient postoperative care, rather than surgical complexity or complication rates

All mastectomy operations were skin-sparing and most reconstructions were performed using a dual-plane implant technique (79.3%). Skin-sparing mastectomy with NAC excision was performed based on National Comprehensive Cancer Network 2025 (NCCN) [16]-consistent indications, including lobular carcinoma with multifocality or patient preference (n = 24), large breast volume with grade III ptosis associated with increased risk of NAC ischemia and suboptimal oncologic safety (n = 53), positive intraoperative frozen-section biopsy of the NAC requiring conversion (n = 17), and tumor-to-nipple distance < 2 cm representing a contraindication to NAC preservation (n = 22). In the delayed reconstruction group (n = 24), LD flap (n = 16, 13.8%) and DIEP flap (n= 8, 6.9%) were the techniques chosen. Symmetrization procedures were performed in 65.5% of cases. Regarding oncologic staging, Stage II disease was most common (48.3%), followed by Stage I (31.0%) and Stage III (20.7%). The main histological subtype was ductal carcinoma (79.3%), with lobular carcinoma accounting for 20.7%. Molecular subtype distribution was Luminal A (n = 51), Luminal B (n = 37), HER2-enriched (n = 17), and triple-negative (n = 11).

In terms of oncological management, 51.7% of patients received post-operative radiotherapy and 65.5% of patients had to undergo chemotherapy. Axillary management was performed in accordance with NCCN Guidelines for Surgical Axillary Staging [16] and was based on initial clinical nodal status and tumor biology, rather than postoperative pathological stage alone. A total of 48 patients underwent primary axillary lymph node dissection (ALND; Berg levels I–II) due to preoperative indications. This group included 11 patients with triple-negative breast cancer, 24 patients with clinically Stage III disease, and 13 patients with Stage IIB (T2N1M0) tumors who had preoperative fine-needle aspiration–proven nodal metastases, received neoadjuvant chemotherapy, and remained clinically node-positive after treatment, warranting ALND.

Among the 68 patients initially treated with sentinel lymph node biopsy (SLNB), 12 required conversion to ALND based on pathological findings, including 7 cases with macrometastatic nodal disease and 5 cases with ≥3 positive sentinel lymph nodes, scenarios in which omission of ALND is not recommended. Consequently, the final distribution consisted of 56 patients (48.3%) treated with sentinel lymph node biopsy and 60 patients (51.7%) who underwent axillary lymphadenectomy.

Most reconstructions were unilateral (79.3%), while 20.7% were bilateral. Indications for bilateral mastectomy and reconstruction included cases with simultaneous contralateral breast cancer or confirmed BRCA mutation carriers, as recommended by the multidisciplinary tumor board. Postoperative complications occurred in 24.1% of patients and were predominantly minor. According to the Clavien–Dindo classification, most complications were classified as **Grade I–II**, consisting mainly of small hematomas and ecchymosis managed conservatively or with bedside interventions. No **Grade III–V** complications were observed, and there were **no cases of implant loss** during the follow-up period. Baseline demographic, clinical, and treatment-related characteristics of the study cohort are presented in Table 2.

### 3.3. Patient-Reported Outcomes: Pre- and Postoperative Comparisons

Of 116 patients, 92 underwent immediate and 24 underwent delayed reconstruction (DIEP n = 8, LD n = 16). Because baseline profiles and technique distributions differed by timing, results are reported separately by cohort: (i) within-group preoperative (preop.) vs. postoperative (postop.) changes in BREAST-Q domains, and (ii) between-technique comparisons within the delayed cohort.

To evaluate changes in patient-reported outcomes over time, we performed paired samples *t*-tests comparing preoperative and postoperative BREAST-Q scores within each cohort (immediate and delayed reconstruction). This approach allowed us to identify whether each surgical pathway was associated with significant improvements or declines across the BREAST-Q domains. Effect sizes (Cohen’s d) were calculated to quantify the magnitude of change, with Levene’s test confirming assumptions of variance where appropriate.

In the immediate reconstruction group (n = 92), psychosocial well-being significantly improved postoperatively, with mean scores rising from 68.30 ± 17.97 preoperatively to 74.74 ± 19.78 postoperatively (t = 6.83, p < 0.001, d = 0.71). Physical well-being also showed a significant increase, from 72.48 ± 14.42 to 75.35 ± 16.62 (t = 2.82, p = 0.006, d = 0.29). Sensation scores decreased significantly after reconstruction, from 85.52 ± 12.17 to 72.26 ± 21.64 (t = −8.43, p < 0.001, d = −0.87). Breast symptoms improved significantly, increasing from 74.35 ± 13.02 to 79.48 ± 15.17 (t = 5.86, p < 0.001, d = 0.61).

**Figure 2:**
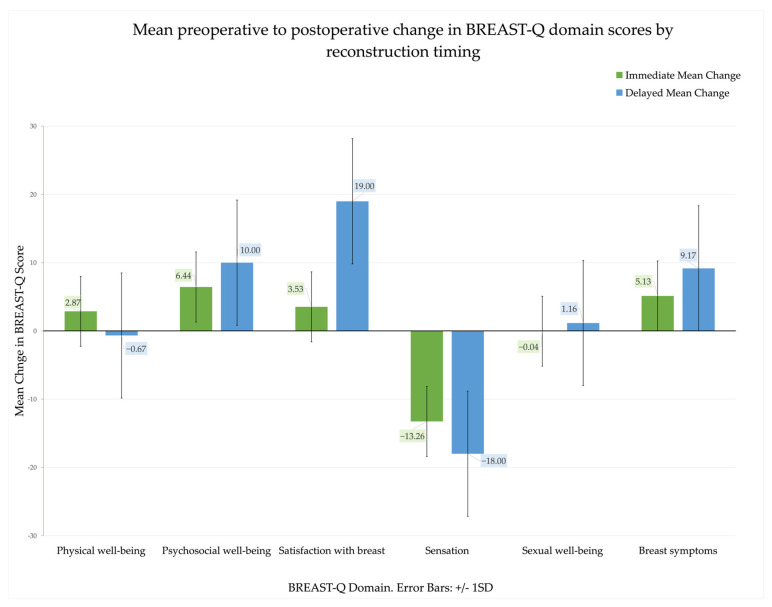
Mean pre- to postoperative change in BREAST-Q domain scores by reconstruction timing. The Y-axis represents mean change in BREAST-Q scores, with positive values indicating improvement and negative values indicating decline. The X-axis lists the evaluated domains. Immediate and delayed reconstruction groups are shown side by side for each domain. Error bars represent ±1 SD.

In the delayed reconstruction group (n = 24), psychosocial well-being improved significantly, rising from 69.83 ± 11.40 preoperatively to 79.83 ± 16.58 postoperatively (t = 4.86, p < 0.001, d = 0.99). Postoperative satisfaction with the breast increased significantly compared with preoperative values (M difference = +19.0 ± 13.8, t(23) = 6.74, p < 0.001, 95% CI [13.17, 24.83]). Bootstrap resampling (1000 iterations) confirmed the robustness of the finding (p < 0.001, 95% CI [14.00, 24.88]). The effect size was large (Cohen’s d = 1.38, Hedges’ g = 1.33), indicating a clinically meaningful postoperative improvement in satisfaction scores. Sensation declined significantly from 78.50 ± 8.06 to 60.50 ± 20.64 (t = −6.36, p < 0.001, d = −1.26). Conversely, breast symptoms significantly improved, with scores increasing from 80.33 ± 11.37 to 89.50 ± 12.58 (t = 10.94, p < 0.001, d = 2.23) [Figure 2, Table 3]. Several observed changes in BREAST-Q domain scores exceeded previously reported minimal clinically important differences (approximately 5–10 points), indicating clinically meaningful effects rather than statistical variation alone.

### 3.4. Between-Group Analyses

**Figure 3:**
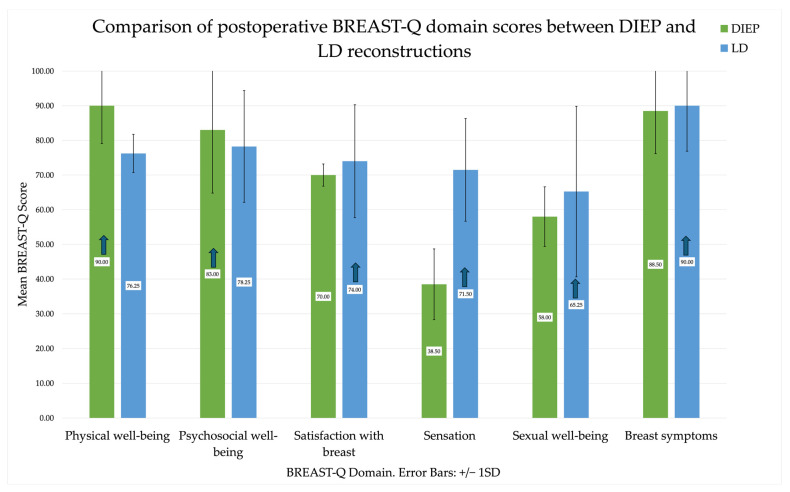
Comparison of postoperative BREAST-Q domain scores between DIEP and LD flap reconstructions in the delayed cohort. Error bars represent ±1 SD. Arrows indicate increase in score.

Independent samples *t*-tests were performed to compare postoperative patient-reported outcomes between LD (n = 16) and DIEP (n = 8) reconstructions in the delayed cohort. No significant differences were observed in psychosocial well-being, sexual well-being, breast satisfaction, or breast symptoms (all p > 0.05). Physical well-being was significantly higher in DIEP patients (M = 90.0, SD = 10.7) compared with LD (M = 76.3, SD = 5.5; p < 0.001, Cohen’s d = 1.82), indicating a large effect. Conversely, sensation scores were significantly lower following DIEP reconstruction (M = 38.5, SD = 10.2) compared with LD (M = 71.5, SD = 14.8; p < 0.001, Cohen’s d = 2.45). Overall, delayed DIEP reconstruction was associated with greater physical well-being but reduced postoperative breast sensation compared with LD flaps [Figure 3].

In the DIEP cohort (n = 8), a paired samples *t*-test revealed a highly significant improvement in abdominal well-being following reconstruction. The mean postoperative abdominal score (M = 10.63, SD = 0.92) was markedly higher compared to the preoperative score (M = 3.88, SD = 0.35), reflecting a mean increase of 6.75 points (95% CI [5.89, 7.62], t(7) = 18.45, p < 0.001). The effect size was very large (Cohen’s d = 6.52, Hedges’ g = 5.79), indicating a substantial and clinically meaningful improvement in abdominal well-being after DIEP reconstruction.

In the LD cohort (N = 16), postoperative scores for the Physical Well-Being: Back and Shoulder module were significantly lower compared with preoperative values (M pre = 82.50, SD = 18.99; M post = 77.75, SD = 23.21). The paired *t*-test indicated a mean decrease of 4.75 points (95% CI [−7.70, −1.80]), t(15) = −3.44, p = 0.004. This corresponds to a large effect size (Cohen’s d = −0.86; Hedges’ g = −0.82, corrected for small sample size). The strong pre–post correlation (r = 0.985, p < 0.001) suggests that individual differences were preserved, but overall patients reported a clinically meaningful deterioration in back and shoulder well-being following LD reconstruction.

To compare the magnitude of change in BREAST-Q domains between immediate and delayed reconstructions, independent samples *t*-tests were applied to the difference scores (postoperative–preoperative). Equality of variances was checked using Levene’s test, and the appropriate statistics were reported accordingly. When comparing immediate (n = 92) and delayed (n = 24) reconstructions, independent samples *t*-tests showed no significant differences in psychosocial well-being (p = 0.062) or sexual well-being (p = 0.336). Physical well-being demonstrated a trend favouring immediate reconstruction (+2.87 vs. −0.67, t = 2.148, p = 0.095 two-sided). Sensory outcomes worsened in both groups, with a more pronounced decline in the delayed cohort, though this was not significant (p = 0.151). Breast-related symptoms improved significantly more in delayed reconstructions (+9.16 vs. +5.13, t = –3.331, p < 0.001).

### 3.5. Predictors of QoL

An ANCOVA was performed to examine the effect of breast symmetrization on post-operative physical well-being, controlling for baseline physical well-being, age, timing, and chemotherapy. The overall model was significant, F(5,110) = 61.65, p < 0.001, explaining 73.7% of the variance in post-operative physical well-being. Symmetrization had a strong and statistically significant effect (F(1,110) = 29.45, p < 0.001, partial η^2^ = 0.211), with patients undergoing symmetrization reporting substantially higher physical well-being scores compared to those without. Baseline physical well-being was also a significant predictor (F(1,110) = 250.96, p < 0.001, partial η^2^ = 0.695). Age, timing, and chemotherapy did not significantly influence outcomes after adjustment.

Pearson correlation analyses revealed strong interrelationships among postoperative BREAST-Q domains, animation deformity, NAC satisfaction, breast symptoms, and the Sensibility–Quality of Life (Sens-QoL) scale. Very strong correlations were observed between psychosocial and sexual well-being (r = 0.83, p < 0.001) and between breast satisfaction, animation deformity, and NAC satisfaction (r = 0.80–0.85, p < 0.001). Physical well-being correlated moderately with psychosocial (r = 0.53) and sexual well-being (r = 0.51), while breast symptoms showed strong associations with physical well-being (r = 0.80) and moderate associations with psychosocial well-being and breast satisfaction (r = 0.54–0.56).

To illustrate the interrelationships among postoperative BREAST-Q domains and related parameters, a correlation heatmap was constructed [Table 4]. The color gradient represents the strength of Pearson’s correlation coefficients (PC), with progressively darker blue shades corresponding to stronger positive associations. Correlations were categorized as very strong (r = 0.80–1.00), strong (0.60–0.79), moderate (0.40–0.59), weak (0.20–0.39), and very weak (0.00–0.19).

A univariate ANCOVA was performed to assess the impact of adjuvant radiotherapy on postoperative breast satisfaction while adjusting for preoperative satisfaction, age, reconstruction timing (immediate vs. delayed), chemotherapy, and reconstruction type. The overall model was highly significant (F(7,108) = 29.29, p < 0.001), explaining 65.5% of the variance in postoperative satisfaction (R^2^ = 0.655; adjusted R^2^ = 0.633). Radiotherapy exerted a strong and statistically significant negative effect on postoperative satisfaction (F(1,108) = 93.15, p < 0.001; partial η^2^ = 0.463), with irradiated patients reporting lower satisfaction scores than non-irradiated patients (64.73 ± 20.72 vs. 74.43 ± 16.72). Importantly, a significant interaction between radiotherapy and preoperative satisfaction was observed (F(1,108) = 91.24, p < 0.001; partial η^2^ = 0.458), indicating that baseline satisfaction differentially influenced postoperative outcomes depending on radiotherapy exposure. Preoperative satisfaction remained a strong independent predictor (partial η^2^ = 0.412; p < 0.001), while chemotherapy and reconstruction type demonstrated smaller but significant effects. Age and reconstruction timing showed non-significant trends. Given the presence of unequal variances between groups (Levene’s test p < 0.001), results should be interpreted with appropriate caution.

Taken together, these findings suggest that radiotherapy exerts a strong and complex influence on postoperative satisfaction with the breast. While patients undergoing radiotherapy generally reported lower satisfaction, the effect varied according to their baseline (preoperative) satisfaction levels, indicating a moderate role of radiotherapy in postoperative patient-reported outcomes.

## 4. Discussion

This study aimed to validate the Romanian version of the BREAST-Q Reconstruction Module and to comprehensively evaluate changes in patient-reported outcomes (PROs) and breast sensation after immediate and delayed reconstruction. Additionally, we examined the influence of reconstructive technique, adjuvant therapy, and symmetrization on postoperative QoL. By combining psychometric validation with clinical outcome analysis, this study provides a contextual overview of patient-reported outcomes after breast reconstruction within the Romanian healthcare setting.

The Romanian BREAST-Q demonstrated excellent internal consistency (Cronbach’s α = 0.947) and strong test–retest reliability (ICC = 0.81–0.92), comparable to other language validations such as the Swedish, Spanish, Danish, and Portuguese versions [17,18,19,20]. The narrow range of “α if item deleted” values confirmed that all 16 items contributed meaningfully to the construct being measured, and the corrected item–total correlations indicated a robust underlying structure. These findings support the stability and reproducibility of the translated instrument for assessing QoL and satisfaction among Romanian breast reconstruction patients, aligning with the psychometric robustness consistently reported in international BREAST-Q studies [21]. The present validation, therefore, ensures cultural and linguistic equivalence, enabling future multinational data comparisons and pooled analyses.

Both immediate and delayed reconstructions resulted in significant postoperative improvements in psychosocial well-being and breast symptom relief, highlighting the reconstructive procedure’s essential role in restoring self-image and alleviating discomfort associated with mastectomy. These findings are consistent with previous studies showing that reconstruction—regardless of timing—enhances psychosocial recovery and confidence [22,23]. Physical well-being improved modestly after immediate reconstruction, likely reflecting faster recovery and less scarring, whereas delayed reconstructions showed stable physical scores, suggesting adaptation following longer intervals since mastectomy.

Breast sensation, however, significantly declined in both groups, particularly in delayed reconstructions (Cohen’s d = −1.26). This pattern mirrors the well-established loss of sensory perception following mastectomy and reconstruction due to nerve transection and limited reinnervation potential [24]. Despite these sensory deficits, the overall improvement in psychosocial outcomes suggests that aesthetic and emotional restoration may compensate for sensory loss in the short term. Notably, delayed reconstructions exhibited greater improvement in breast symptom scores, possibly reflecting resolution of tightness or scar-related discomfort after previous mastectomy, as well as improved soft-tissue pliability following autologous flap coverage.

Within the delayed cohort, comparison between DIEP and LD flaps revealed clear domain-specific contrasts. DIEP reconstructions were associated with significantly higher postoperative physical well-being but markedly reduced breast sensation compared with LD flaps. The higher physical well-being scores observed in patients undergoing DIEP reconstruction may be related to the absence of an implant and preservation of the pectoralis major muscle, resulting in reduced chest wall discomfort and avoidance of animation deformity; however, given the small number of DIEP cases in our cohort, these findings should be interpreted as exploratory and consistent with previously reported trends rather than definitive evidence [25]. Conversely, the poorer sensory recovery in DIEP flaps aligns with the greater nerve disruption inherent in total autologous reconstruction and the absence of direct sensory neurotization unless specifically performed. In contrast, LD flaps—by retaining a portion of the thoracodorsal nerve and partial innervation—may allow limited spontaneous reinnervation of the overlying skin envelope. However, this comes at the cost of donor-site morbidity, as evidenced by the significant decline in back and shoulder well-being (p = 0.004, d = −0.86) observed in our cohort and in the literature [26]. Our findings should be interpreted in the context of the existing literature. While meta-analyses and large cohort studies have reported superior BREAST-Q outcomes following DIEP flap reconstruction compared with LD flaps—particularly in breast satisfaction and physical well-being [27,28,29]—such differences were not consistently observed in our cohort. In our analysis, DIEP and LD reconstructions demonstrated largely comparable BREAST-Q scores across most domains, apart from higher physical well-being and lower sensory scores among DIEP patients. This apparent divergence from international datasets is most likely attributable to the small size of the delayed autologous reconstruction subgroup (n = 24; LD n = 16, DIEP n = 8), in which interindividual variability may outweigh technique-specific effects. Accordingly, these findings are considered exploratory and should not be interpreted as contradicting established evidence.

Symmetrization emerged as a significant independent predictor of postoperative physical well-being (partial η^2^ = 0.211). Patients who underwent contralateral symmetrization reported greater physical comfort and improved body balance, findings consistent with previous reports highlighting the role of breast symmetry in promoting musculoskeletal comfort, posture, and positive body perception [30]. Multiple studies have shown that performing contralateral symmetrization at the time of breast reconstruction results in higher satisfaction with breast appearance, improved psychosocial and sexual well-being, and overall better QoL when assessed with validated tools such as the BREAST-Q and SF-36 [31,32,33]. Immediate symmetrization has also been associated with greater breast satisfaction compared with delayed procedures, without an increase in complication or revision rates [34]. The long-term benefits extend beyond aesthetics, as patients who undergo symmetrization report better physical, social, and psychological outcomes at six months and beyond, with notable improvements in symmetry, clothing fit, and self-image [31,32,35]. Moreover, in DIEP flap reconstruction, the addition of symmetrization and other complementary procedures—such as nipple-areolar reconstruction—has been shown to further enhance satisfaction, particularly within psychosocial domains [35]

Adjuvant radiotherapy was identified in our analysis as a strong negative predictor of postoperative satisfaction with the breast (partial η^2^ = 0.463), consistent with its well-documented detrimental effects on capsular contracture, fibrosis, and aesthetic distortion [36]. Multiple large meta-analyses and cohort studies have similarly demonstrated that radiotherapy markedly reduces postoperative satisfaction and QoL among patients undergoing implant-based reconstruction, with significantly lower BREAST-Q scores across satisfaction, psychosocial, and physical well-being domains, as well as higher rates of complications, reoperations, and reconstruction failure [37,38,39]. This effect is both statistically and clinically meaningful, with irradiated patients reporting satisfaction scores approximately **7–9 points lower**, exceeding the minimal clinically important difference and associated with less favorable aesthetic outcomes [39,40,41,42]. In contrast, this negative impact appears attenuated in autologous reconstruction, where satisfaction and quality-of-life scores remain higher and complication rates lower than in implant-based procedures [43,44].

Across reconstructive modalities, autologous reconstruction—particularly procedures such as the DIEP flap—has consistently been associated with higher satisfaction with the breast, overall outcome, psychosocial well-being, and sexual well-being compared with implant-based reconstruction, both in the short and long term [45,46,47]. These benefits extend beyond aesthetics, encompassing improved tactile quality, natural softness, and a stronger sense of bodily integrity [48,49]. Importantly, higher satisfaction after autologous reconstruction remains clinically meaningful after adjustment for confounders and appears durable over time, with longitudinal studies demonstrating sustained or improving quality-of-life outcomes [50,51,52].

In contrast, while implant-based reconstruction substantially improves QoL compared with mastectomy alone, satisfaction scores tend to be lower than those reported after autologous procedures, particularly in domains related to breast appearance, feel, and naturalness [45,46,51,53]. Nevertheless, implant-based methods are associated with comparable physical well-being of the chest and typically involve shorter operative times, faster recovery, and avoidance of donor-site morbidity [51,52,53,54,55].

Findings from multiple meta-analyses and large multicenter cohort studies reinforce these patterns. Across studies using validated patient-reported outcome measures such as the BREAST-Q, autologous reconstruction demonstrates a mean difference of approximately 6–10 points higher on breast satisfaction scales (0–100 range) compared with implant-based methods—well above the minimal clinically important difference [45,46,56,57].

Correlation analysis in our cohort revealed strong interrelationships between psychosocial and sexual well-being (r = 0.83) and between satisfaction with the breast, NAC satisfaction, and animation deformity (r = 0.80–0.85). These associations support the conceptual coherence of the BREAST-Q framework, in which body image restoration and aesthetic perception are central to psychosocial recovery. This finding aligns with extensive evidence demonstrating that NAC reconstruction—whether achieved through surgical techniques, medical tattooing, or prosthetic options—significantly enhances patient satisfaction with breast appearance, improves body image, and increases overall contentment with the reconstructive process [58,59,60,61]. Several studies have also linked NAC reconstruction to higher psychosocial and sexual well-being scores, as measured by validated tools such as the BREAST-Q, with consistent benefits observed across both implant-based and autologous reconstructions [58,59,60,61,62]. Importantly, the specific technique appears to influence the degree of satisfaction: nipple-sparing mastectomy (NSM) typically yields the highest scores, while local flap reconstructions (particularly C–V flaps) and three-dimensional tattooing also provide favorable and durable results when NSM is not feasible [63,64,65]. For patients unable or unwilling to undergo additional surgery, NAC prostheses offer a non-invasive alternative associated with high patient-reported satisfaction [64,66]. Collectively, these findings highlight the crucial aesthetic and psychological role of NAC reconstruction in promoting a sense of wholeness and completeness after breast reconstruction, underscoring its impact on both physical appearance and emotional recovery.

Breast sensation plays a fundamental role in shaping patient satisfaction and overall QoL after reconstruction. Numerous studies have demonstrated that improved sensory recovery of the breast and NAC is strongly associated with higher patient-reported satisfaction, particularly within psychosocial and sexual well-being domains [66,67,68]. Autologous reconstruction techniques, such as the DIEP flap with neurotization, tend to achieve superior sensory outcomes compared with implant-based methods, contributing to greater perceived naturalness and higher satisfaction scores [67,68,69,70]. Furthermore, the incorporation of neurotization—through direct nerve coaptation or nerve grafting—has been shown to significantly enhance sensory recovery, translating into measurable improvements in QoL and body image [71,72]. Objective assessments of tactile and pressure thresholds using standardized tests, such as Semmes–Weinstein monofilaments, correlate closely with BREAST-Q scores for physical well-being of the chest, as well as psychosocial and sexual well-being [71,73]. Conversely, persistent sensory loss or the absence of neurotization is consistently linked to lower QoL, with adverse effects on body image, confidence, clothing comfort, and sexual function [25,68,69]. Collectively, these findings emphasize that sensory restoration is not merely a functional consideration but a key determinant of postoperative satisfaction and emotional recovery, supporting the integration of nerve-preserving and neurotization strategies in modern reconstructive practice.

Minimally invasive breast surgery techniques, including video-assisted approaches, have been shown to enhance postoperative patient-reported outcomes by improving flap quality and limiting tissue trauma, factors that are closely linked to postoperative comfort, physical well-being, and overall aesthetic satisfaction in reconstructed breasts [74].

When contextualized within the European literature, patient-reported outcomes observed in this Romanian cohort are broadly comparable to those reported in Western and Central European populations using the BREAST-Q. Similar patterns have been described across Europe, with reconstruction associated with improvements in psychosocial well-being and satisfaction, while adjuvant radiotherapy consistently exerts a negative impact on patient-reported outcomes [51]. The validation of the Romanian BREAST-Q facilitates the inclusion of Romanian patients in international outcome registries and multicenter trials, promoting standardized assessment of patient-reported metrics across cultural contexts. In Romanian clinical practice, the validated BREAST-Q Reconstruction Module can be used to objectively monitor patient-reported outcomes after breast reconstruction, guide preoperative counseling regarding expected physical well-being, sensation, and satisfaction, and identify patients—particularly those undergoing radiotherapy or delayed reconstruction—who may benefit from closer follow-up and tailored supportive interventions.

This study has several limitations. It was conducted in a single tertiary center with a moderate overall sample size, and the number of patients undergoing autologous reconstruction—DIEP and LD flaps—was relatively small, limiting the generalizability of autologous outcome analyses. In addition, the absence of objective sensory testing and the relatively short follow-up period of one year may restrict the interpretation of long-term sensory recovery and quality-of-life outcomes. Future research should incorporate objective sensory testing methods (e.g., monofilament or thermal threshold assessments) and longitudinal designs extending beyond one year to capture late-phase adaptation and reinnervation. Additionally, multicenter collaboration could enhance representativeness and enable stratified analyses across reconstruction techniques.

## 5. Conclusions

The Romanian version of the BREAST-Q Reconstruction Module demonstrated excellent reliability and validity for assessing patient-reported quality of life after breast reconstruction. Within the limitations of this single-center cohort, exploratory analyses indicated improvements in psychosocial well-being and breast symptoms following reconstruction, while reduced sensation was observed during the first postoperative year. Autologous reconstruction and symmetrization were associated with greater physical comfort, whereas radiotherapy was negatively associated with satisfaction. These findings warrant confirmation in larger, multicenter studies with longer follow-up.

## Figures and Tables

**Table 1:** Internal Consistency of the Romanian BREAST-Q Questionnaire (n = 16 items).

Domain/Statistic	Cronbach’s α	Corrected Item–Total Correlation (Range)	α if Item Deleted (Range)
Overall Scale	0.947	0.362–0.971	0.938–0.952

**Table 2:** Characteristics of the study population (n = 116).

Characteristic	n = 116	%
Reconstruction type		
● Dual-plane implant	92	79.3
● LD flap	16	13.8
● DIEP flap	8	6.9
Timing of reconstruction		
● Immediate	92	79.3
● Delayed	24	20.7
Symmetrization	76	65.5
Stage		
● Stage I	36	31.0
● Stage II	56	48.3
● Stage III	24	20.7
Histological type		
● Ductal	92	79.3
● Lobular	24	20.7
Molecular subtype		
● Luminal A	51	43.9
● Luminal B	37	31.8
● HER enriched	17	14.6
● Triple-negative	11	9.4
Chemotherapy	76	65.5
Radiotherapy	60	51.7
Unilateral reconstruction	92	79.3
Bilateral reconstruction	24	20.7
Complications	28	24.1
Smoking	32	27.6
Arterial Hypertension	42	36.2
Diabetes mellitus	36	31.0
Marital status		
● Married	96	82.8
● Not married	12	10.3
● Widow	4	3.4
● Partner	4	3.4

**Table 3:** Paired *t*-test Results Immediate vs. Delayed Reconstruction.

Domain	Immediate PREOP (Mean ± SD)	Immediate POSTOP (Mean ± SD)	Immediate p-Value	Immediate Effect Size (g)	Delayed PREOP (Mean ± SD)	Delayed POSTOP (Mean ± SD)	Delayed p-Value	Delayed Effect Size (g)
**Psychosocial well-being**	68.30 (17.97)	74.74 (19.78)	<0.001	0.71	69.83 (11.40)	79.83 (16.58)	<0.001	0.96
**Sexual well-being**	60.13 (17.59)	60.09 (20.17)	0.950	−0.01	61.67 (19.64)	62.83 (20.72)	0.273	0.22
**Physical well-being**	72.48 (14.42)	75.35 (16.62)	0.006	0.29	81.50 (9.40)	80.83 (9.91)	0.612	−0.10
**Satisfaction with breast**	65.04 (23.15)	68.57 (20.69)	0.135	0.16	58.83 (8.55)	77.83 (12.16)	<0.001	1.33
**Sensation**	85.52 (12.17)	72.26 (21.64)	<0.001	−0.87	78.50 (8.06)	60.50 (20.64)	<0.001	−1.26
**Breast symptoms**	74.35 (13.02)	79.48 (15.17)	<0.001	0.61	80.33 (11.37)	89.50 (12.58)	<0.001	2.16

**Table 4:** Correlation heatmap showing associations among postoperative BREAST-Q domains, animation deformity, NAC satisfaction, breast symptoms, and Sens–QoL. Colour intensity reflects correlation strength (dark blue = very strong, light blue = weak).

		Psychosocial Well-Being-POSTOP	Sexual Well-Being POSTOP	Physical Well-Being-POSTOP	Satisfaction with Breas-POSTOP	Animation Deformity	NAC Satisfaction	Sensation-POSTOP	Breast Symptoms-POSTOP	Sensation QOL
Psychosocial well-being-POSTOP	PC	1	0.829 **	0.528 **	0.783 **	0.742 **	0.708 **	0.331 **	0.542 **	0.389 **
	p		<0.001	<0.001	<0.001	<0.001	<0.001	<0.001	<0.001	<0.001
Sexual well-being POSTOP	PC	0.829 **	1	0.506 **	0.845 **	0.667 **	0.588 **	0.446 **	0.491 **	0.244 **
	p	<0.001		<0.001	<0.001	<0.001	<0.001	<0.001	<0.001	0.008
Physical well-being-POSTOP	PC	0.528 **	0.506 **	1	0.556 **	0.439 **	0.555 **	0.235 *	0.799 **	0.227 *
	p	<0.001	<0.001		<0.001	<0.001	<0.001	0.011	<0.001	0.014
Satisfaction with breast-POSTOP	PC	0.783 **	0.845 **	0.556 **	1	0.845 **	0.804 **	0.592 **	0.561 **	0.298 **
	p	<0.001	<0.001	<0.001		<0.001	<0.001	<0.001	<0.001	0.001
Animation Deformity	PC	0.742 **	0.667 **	0.439 **	0.845 **	1	0.802 **	0.736 **	0.515 **	0.403 **
	p	<0.001	<0.001	<0.001	<0.001		<0.001	<0.001	<0.001	<0.001
NAC Satisfaction	PC	0.708 **	0.588 **	0.555 **	0.804 **	0.802 **	1	0.421 **	0.649 **	0.145
	p	<0.001	<0.001	<0.001	<0.001	<0.001		<0.001	<0.001	0.224
Sensation-POSTOP	PC	0.331 **	0.446 **	0.235 *	0.592 **	0.736 **	0.421 **	1	0.177	0.061
	p	<0.001	<0.001	0.011	<0.001	<0.001	<0.001		0.058	0.514
Breast symptoms-POSTOP	PC	0.542 **	0.491 **	0.799 **	0.561 **	0.515 **	0.649 **	0.177	1	0.302 **
	p	<0.001	<0.001	<0.001	<0.001	<0.001	<0.001	0.058		<0.001
Sensation QOL	PC	0.389 **	0.244 **	0.227 *	0.298 **	0.403 **	0.145	0.061	0.302 **	1
	p	<0.001	0.008	0.014	0.001	<0.001	0.224	0.514	<0.001	

*. Correlation is significant at the 0.05 level (2-tailed). **. Correlation is significant at the 0.01 level (2-tailed).

## Data Availability

The raw data supporting the conclusions of this article will be made available by the authors on request.

## Abbreviations

The following abbreviations are used in this manuscript:

QoL	Quality of life
SD	Standard Deviation
NAC	Nipple areola complex
LD	Latissimus Dorsi
DIEP	Deep Inferior Epigastric Perforator
HTA	Arterial Hypertension
NCCN	National Comprehensive Cancer Network
ALND	axillary lymph node dissection
SLNB	sentinel lymph node biopsy
